# A BAC end view of the *Musa acuminata *genome

**DOI:** 10.1186/1471-2229-7-29

**Published:** 2007-06-11

**Authors:** Foo Cheung, Christopher D Town

**Affiliations:** 1 J Craig Venter Institute, 9712 Medical Center Drive, Rockville, MD 20850 USA

## Abstract

**Background:**

*Musa *species contain the fourth most important crop in developing countries. Here, we report the analysis of 6,252 BAC end-sequences, in order to view the sequence composition of the *Musa acuminata *genome in a cost effective and efficient manner.

**Results:**

BAC end sequencing generated 6,252 reads representing 4,420,944 bp, including 2,979 clone pairs with an average read length after cleaning and filtering of 707 bp. All sequences have been submitted to GenBank, with the accession numbers DX451975 – DX458350. The BAC end-sequences, were searched against several databases and significant homology was found to mitochondria and chloroplast (2.6%), transposons and repetitive sequences (36%) and proteins (11%). Functional interpretation of the protein matches was carried out by Gene Ontology assignments from matches to *Arabidopsis *and was shown to cover a broad range of categories. From protein matching regions of *Musa *BAC end-sequences, it was determined that the GC content of coding regions was 47%. Where protein matches encompassed a start codon, GC content as a function of position (5' to 3') across 129 bp sliding windows generates a "rice-like" gradient. A total of 352 potential SSR markers were discovered. The most abundant simple sequence repeats in four size categories were AT-rich. After filtering mitochondria and chloroplast matches, thousands of BAC end-sequences had a significant BLASTN match to the *Oryza sativa *and *Arabidopsis *genome sequence. Of these, a small number of BAC end-sequence pairs were shown to map to neighboring regions of the *Oryza sativa *genome representing regions of potential microsynteny.

**Conclusion:**

Database searches with the BAC end-sequences and *ab initio *analysis identified those reads likely to contain transposons, repeat sequences, proteins and simple sequence repeats. Approximately 600 BAC end-sequences contained protein sequences that were not found in the existing available *Musa *expressed sequence tags, repeat or transposon databases. In addition, gene statistics, GC content and profile could also be estimated based on the region matching the top protein hit. A small number of BAC end pair sequences can be mapped to neighboring regions of the *Oryza sativa *representing regions of potential microsynteny. These results suggest that a large-scale BAC end sequencing strategy has the potential to anchor a small proportion of the genome of *Musa acuminata *to the genomes of *Oryza sativa *and possibly *Arabidopsis*.

## Background

Until novel technologies that will enable extremely low-cost genomic DNA sequencing are developed, funding bodies are very selective when choosing new plant genomes to sequence. Current technologies are only able to produce the sequence of a mammalian-sized genome of the desired data quality for $10 to $50 million or more. The initial goal of many genome projects is often to gain a glimpse of the genome of interest at a low cost and in an effective manner. In plants there is often some advantage in leveraging the finished genomes of *Arabidopsis thaliana *and *Oryza sativa *through comparative genomics. *A. thaliana *was chosen as model for the dicotyledons due to its small genome size (125 Mb) [1] and rice [2] (*O. sativa*) was the first cereal and monocot to be sequenced [3].

*Musa *species (bananas and plantains) comprise very important crops in sub-Saharan Africa, South and Central America and much of Asia. The *Musa *species *Musa acuminata *(AA genome) and *Musa balbisiana*, (BB genome), both with 2n = 22 chromosomes represent the two main progenitors of cultivated banana varieties. The haploid genome of *Musa *species was estimated as varying between 560 to 800 Mb in size [4-6], over four times larger than that of the model plant *A. thaliana *(125 Mb) [7] and over 30% larger than that of *O. sativa *(390 Mb) [2].

Comparative genomics in the monocots have focused on the extent of synteny between closely-related species of monocots belonging to the family of Poaceae [8]. Extensive micro and macro synteny has been shown between *O. sativa*, barley, maize and wheat [9,10] and the degree of conservation often varies between different chromosomal locations. Synteny between distantly related plants is more bioinformatically challenging to elucidate and probably occurs less frequently.

In order to understand the sequence content and sequence complexity of the *Musa *genome, it is necessary to sequence a large number of randomly selected clones that are representative of the entire genome. An alternative approach is to end-sequence a large number of Bacterial Artificial Chromosomes (BACs) randomly selected from a BAC library [11]. This latter approach does not provide a truly random sampling of the genome since regions in which the restriction site for the particular enzyme used for library construction is under-represented will also be under-represented. Nevertheless, BAC end sequencing does provide a quasi-random sampling of the genome and carries with it the advantage that BAC clones that appear to contain targets of interest provide excellent material for other analyses such as fluorescent *in situ *hybridization (FISH) to metaphase or pachytene chromosomes or in depth sequencing for gene discovery. A large collection of BAC end-sequences (BES) is also an essential component of a genome sequencing project. Here, we examined whether *Musa *BES can lead to insights into the *Musa *genome composition using bioinformatic comparisons to protein, repeat, expressed sequence tags (ESTs) and other databases. From the BES, we investigate the *Musa *gene density, GC content, protein and SSR content and putative comparative-tile BACs that represents potential regions of microsynteny between the *O. sativa *and *Musa *species.

## Results and discussion

Sequence searches, simple sequence repeats, GC profiling and protein discovery will be discussed first, followed by an analysis of genome mapping to *O.sativa *and *A. thaliana *to identify comparative tile BACs from the *Musa *library that will be likely collinear (i.e. showed microsynteny).

### BAC end sequencing

End sequencing of BACs from a HindIII BAC library constructed from leaves of the wild diploid 'Calcutta 4' clone [12], generated 6,252 high quality reads with an average length of 707 nucleotides, giving a total length of ~ 4.4 Mb that included 2,979 paired end reads (Table 1). All sequences have been submitted to GenBank, with the accession numbers DX451975 – DX458350.

**Table 1:** Sequence statistics of the *Musa *BES

Total # sequences	6,252
Total base count (bp)	4,420,944
Minimum length (bp)	101
Average length (bp)	707
Maximum length (bp)	1,007

### Database sequence searches

**Figure 1:**
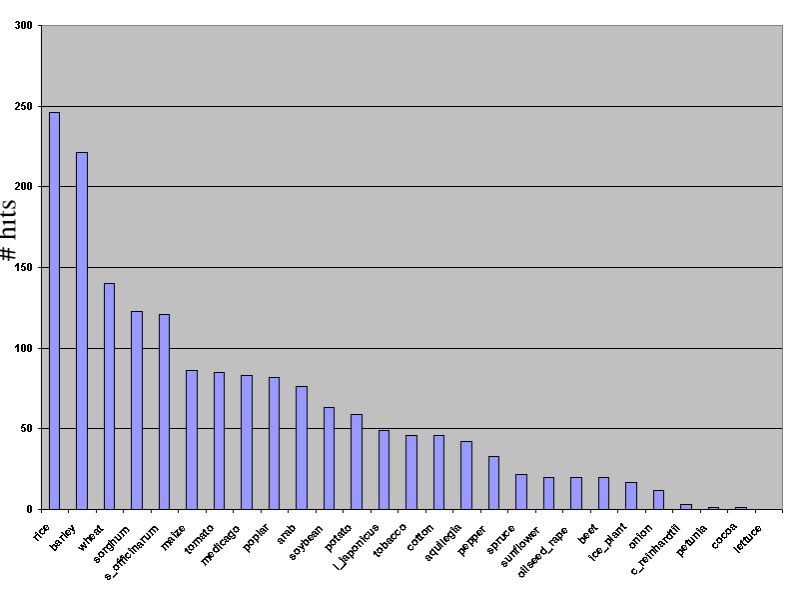
Number of *Musa *BES containing hits to The TIGR Plant Gene Indices using blat.

Comparison of the BES with the TIGR non-identical amino acid database revealed that 11% of the sequences contained "genic" regions by virtue of good matches, excluding transposons/repeats (36%). Using a stringent threshold of 1e-5, 80% identity and 80% coverage resulted in 2.6% BES matches to chloroplast/mitochondria (Table 2). Of the protein matches, the top BLAST match in over 50% of cases was to *O.sativa *and in 30% to *A. thaliana *proteins, consistent with the closer relatedness between *Musa *and *O. sativa *when compared to *Musa *and *A. thaliana*. This is also consistent with matches to the TIGR Plant Gene Indices where the highest level of homology was shown to *O. sativa *followed by barley, wheat and other monocots (Figure 1). Of the BES analysed, 36% were found to contain sequences homologous to transposable elements or repeats. The majority of transposable elements belonged to the Ty1 copia type (742) followed by the Ty3 gypsy (211) types of retrotransposons (Table 2) consistent with previous data that class I retrotransposons contributing to most of the nucleotide [13] and from studies using papaya BAC end sequences.

**Table 2:** Sequence similarity search results

Database	Number of hits (%)
Mitochondria + Chloroplast	162 (2.6)
Transposon + Repeats	2,291 (36.6)
TIGR protein database	686 (11)
**Total number of BAC ends**	**3,139 (50.2)**

We also found 111 matches to miniature inverted repeat transposable elements (MITEs), the most abundant being adh-11-like (46), followed by adh type D-like (22) and adh type G-like (12). Gene density predictions calculated from the number BES with protein matches (686) at E = 1e-15 estimates the presence of a gene every 6.4 kb (Table 3) which is consistent with previous gene density studies from one *Musa *BAC studied [14]. In contrast, a second BAC from the same study gave a gene density of a gene in every 10 kb, however upon closer examination one half of the BAC consisted of transposon related genes while the other half was non-transposon related. The discrepancy between the data suggests that the gene organization resembling Gramineae where genes are clustered in gene-rich regions separated by gene-poor DNA containing abundant transposons. In comparison with other plant genomes, gene density appears to be similar to reports for the automatic annotation for *O. sativa *of 6.2 kb per gene [15] and different from *A. thaliana *with 4.5 kb per gene [6].

**Table 3:** Summary of transposon content

Transposon Type	Number of BES
Ty3-gypsy	211
Ty1-copia	742
LINE element	12
MUDR element	2
Athila	4
Endovir	31
MITES	111

### Functional annotation

**Figure 3:**
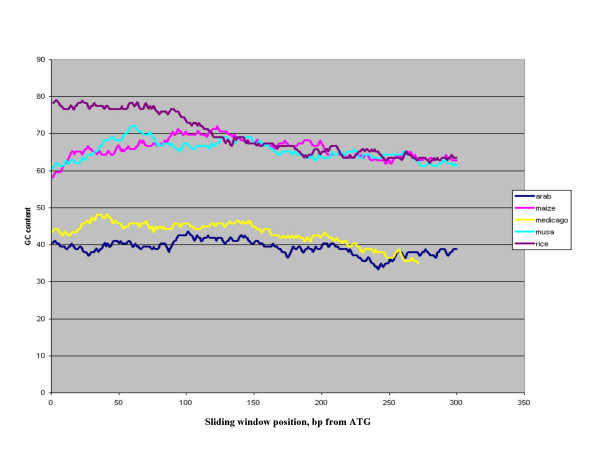
Mean GC content as a function of position (5' to 3') across 129 bp sliding windows.

Gene Ontology (GO) is a controlled vocabulary of functional terms that allows consistent annotation of gene products [16]. In order to assign putative functional roles to the *Musa acuminata *sequences, we used the GO assignments of the *A. thaliana *proteome [16]. Among the 686 BES that did not contain a match to the repeat or transposon databases but contained a match the TIGR comprehensive protein database, 664 had matches to *A. thaliana *proteins and were given GO assignments based on the top matches. The genes are shown to cover a broad range of GO categories (Figure 3).

### GC profile

**Figure 2:**
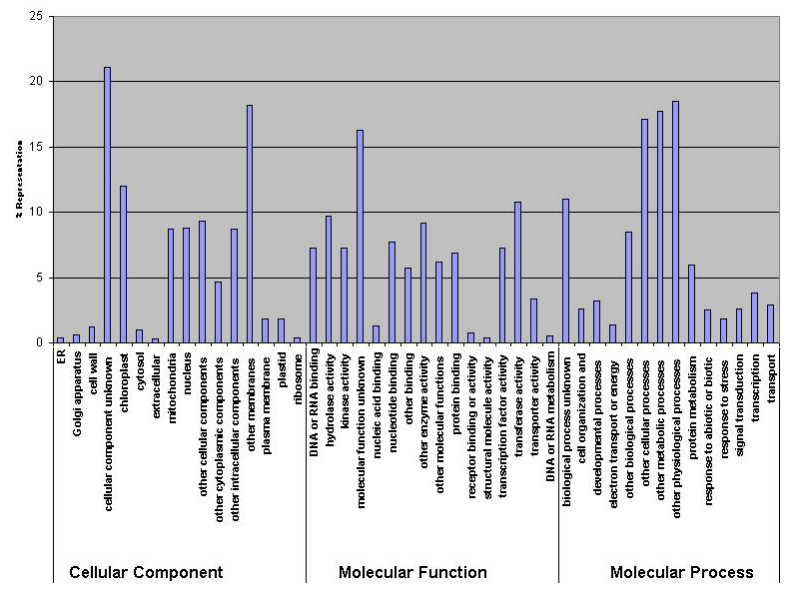
Gene Ontology assignments for *Musa *BES.

GC profiling was performed on the matching region between the BES and the top protein hit. Any BES not containing a match from the start codon was excluded. In parallel, a similar study was carried out for *A. thaliana*, *O. sativa*, maize and *Medicago truncatula *BES (Figure 2). *A. thaliana *and *M. truncatula *showed similar GC content along the entire coding sequence. In most cases *Musa*, *O. sativa *and maize showed a higher GC value at the 5' end within the first 150 bp from the predicted start site, which gradually decreased towards the 3' end. This result is consistent from previous reports where it has been shown that grasses have high mean GC content and asymmetrical distributions, while the eudicots have lower GC content and more symmetrical distributions [17,18]

### GC content

The GC content for organisms varies between the genomic, intron and exon regions and can be as low as 22% (*Plasmodium falciparum*) to more than 70% (*Zea mays*). GC content was determined on the matching region between the BES and the top protein hit. The mean GC content of all BES was 39% and coding sequence GC content was 47% consistent with previous studies which was shown to have an overall GC content to be 38% and within exons to be 49% based on 2 BACs [14]. This and the previous section have shown that BES with protein matches can allow GC content and GC profiling to be calculated with some degree of accuracy. Further confirmation using a larger dataset was carried out using ESTs,- 2,280 *Musa *ESTs [19] was downloaded from GenBank, clustered and assembled to give 1,123 unique sequences of which 179 were contigs. The unique sequences generated 1,056 potential open reading frames containing an average GC content of 51%. These results are consistent with previous studies on GC content within monocots and dicots [17].

### Simple sequence repeats

Simple sequence repeats (or microsatellites) are a class of molecular markers that are often polymorphic and are widely used for generating genetic maps [20]. A total of 352 potential SSR markers were discovered within the BAC end-sequences (Table 4). The most abundant SSRs in all four size categories were AT-rich. This is in agreement with previous reports of microsatellite abundance in other species: poly(AT)/(TA) and AT-rich trinucleotide repeats were the most abundant repeats of their class in *A. thaliana *and in yeast [21]. Similar to observations for *Rosaceae *ESTs [22], dinucleotide repeats represent the most abundant of the four microsatellite classes. None of the SSRs present in this study has been reported previously and no matches were found with previous identified Musa SSRs [23,24].

**Table 4:** Distribution of SSRs

Repeats	Total
A/T	4
C/G	1
AC/GT	16
AG/CT	111
AT/AT	130
AAC/GTT	2
AAG/CTT	21
AAT/ATT	18
ACC/GGT	1
ACG/CTG	3
ACT/ATG	1
AGG/CCT	4
AGT/ATC	3
CCG/CGG	1
AAAC/GTTT	1
AAAG/CTTT	1
AAAT/ATTT	12
AACG/CTTG	1
AACT/ATTG	1
AATC/AGTT	2
AATG/ACTT	1
ACAT/ATGT	4
ACCT/ATGG	1
ACGC/CGTG	1
ACGT/ATGC	1
AGAT/ATCT	1
AGCG/CGCT	1
AACCG/CTTGG	1
AATAT/ATATT	1
AATGG/ACCTT	1
AGAGC/CGTCT	1
ACATAT/ATATGT	2
ACTCGG/AGCCTG	1
AGATAT/ATATCT	1

### *Musa *BAC end tiling on the *O. sativa *and *A. thaliana *genome

For a relatively uncharacterized species where there may be synteny with some chromosomal regions of well sequenced model species, high throughput BAC end sequencing offers the potential to 'tile' the genome of the uncharacterized species onto to that of the sequenced species. BES mapping to *O. sativa *and *A. thaliana *were carried out in order to further characterize our BAC library and to test whether a BAC end sequencing approach might be effective for *Musa *in the manner described above. When the Musa BESs were compared to *O. sativa *genome sequence (TIGR *O. sativa *assembly version 4.0 [15]), 2,646 had a significant hit to *O. sativa *with percent identities ranging from 58% – 98% for top matches. These hits included 593 paired reads of which a total of 55 pairs were shown to have the top blast hit to the same chromosome after filtering for homology to mitochondrial and chloroplast matches. Eight BES pairs were shown to have similarity matches of *O. sativa *sequence with a span of 100 to 500 Kb (Table 5). When the Musa BESs were compared to *A. thaliana genome*[7], 2,177 had matches, with percent identities ranging from 54% – 98% for top matches. Amongst the 2,177 hits, 403 BES pairs had a significant BLAST match (both members of the pair) to *A. thaliana *genome sequence of which a total of 36 pairs were shown to have the top blast hit to the same chromosome after filtering for homology to mitochondria and chloroplast matches. Although a small number of BES pairs were shown to have similarity matches of *A. thaliana *sequence with a span of 22 to 500 kb none of them were found in the proper orientation which may represent localised inversions.

**Table 5:** Musa BAC end tiling on the O. sativa genome

Reads	Clone	Coordinates (bp)	Span (bp)	*O. sativa *chromosomal location
MAMAC34TF/MAMAC34TR	MAMAC34	8780081-9289856	509,775	4
MAMA945TF/MAMA945TR	MAMA945	25025509-24588294	437,215	8
MAMAH84TF/MAMAH84TR	MAMAH84	2641587-2209898	431,689	2
MAMAE66TF/MAMAE66TR	MAMAE66	19669753-19399800	269,953	10
MAMA777TF/MAMA777TR	MAMA777	20538799-20725362	186,563	8
MAMA481TF/MAMA481TR	MAMA481	23108313-22926983	181,330	5
MAMAZ34TF/MAMAZ34TR	MAMAZ34	30878620-30754915	123,705	3
MAMAA26TF/MAMAA26TR	MAMAA26	34290570-34168654	121,916	4

*Musa *BACs that fulfil the criteria of having top blast hits to the same chromosome and having no homology to mitochondria and chloroplast were deemed candidate putative comparative-tile-BACs, and potentially represent regions of highly conserved gene content and organization. The predicted size of the *Musa *BACs (and thus the distance between the end-sequences) was compared to the span by which the paired matches are separated in the *O. sativa *and *A. thaliana *genomes respectively. Separations in the *Musa *BES matches that exceeded our arbitrary cut off of 500 Kb, may represent expansions of the syntenic regions and due to rearrangements during the evolution of the two genomes.

## Conclusion

In this study, 2 major ideas were examined. Firstly, that Musa BES can lead to insights into the Musa genome with specific reference to gene density, GC content, protein and SSR discovery; and secondly, that the sequences can be used to identify regions of potential microsynteny between Musa and other species. The BAC end-sequences were shown to contain homology to proteins, expressed sequence tags, transposons, repeat sequences and to be useful for simple sequence repeat identification and estimation of gene statistics and GC content. Proteins encoded in these BES were shown to cover a broad range of GO categories. Although there is only limited microsynteny between Musa and O. sativa, the results suggest that a large-scale BAC end sequencing strategy has the potential to anchor at least a small portion of the genome of Musa onto that of the sequence of the O. sativa. Large-scale BAC end sequencing would show whether there are more regions of microsynteny between the reference genome and the genome of interest and if there was support for whole genome sequencing due to unique gene features and genome characteristics. BAC end data would be one useful indicator along with existing EST or genomic sequences for funding bodies to use when selecting new plant genomes to sequence and assess the potential of leveraging the finished genomes of A. thaliana and O. sativa through comparative genomics. We expect that a similar analysis using other plant or animal species would provide insights into the genome in a very cost effective and efficient manner through database searches and synteny to model species.

## Methods

### BAC end sequencing

The BES were generated from a *Musa *bacterial artificial chromosome (BAC) library constructed from leaves of the wild diploid 'Calcutta 4' clone (*Musa acuminata *subsp. Burmannicoides 2n = 2 × = 22) with an average insert size of 100 kb [12].

DNA template was prepared in 384-well format by a standard alkaline lysis method. End sequencing was performed using Applied Biosystems (ABI) Big Dye terminator chemistry and analyzed on ABI 3730 xl machines. Base calling was performed using TraceTuner and sequences were trimmed for vector and low quality sequences using Lucy [25].

### BAC end database searches

Sequences were compared to all entries in the TIGR Plant Gene Indices [26] using blat and to the TIGR non-identical amino acids database that contains non-identical protein data from a number of databases including GenBank, RefSeq and Uniprot using blastx (cut-off value 1e-5). The BAC end-sequences were also compared with repetitive sequences in the TIGR Repeat Database [27] and an in-house transposon database using blastx with a cut-off value of 1e-5. The BAC end-sequences were compared with the TIGR rice genome sequence assembly and the *A. thaliana *genome sequence from TAIR using blastn with a cut-off value of 1e-10. To identify comparative tile BACs from the *Musa *library that were likely collinear (i.e. showed microsynteny) with the reference genomes, the searches against the *Musa *genomic sequence were parsed for the top pair of BES for which both ends had the highest significant match to a stretch of *O. sativa *or *A. thaliana *sequence and where the two regions on the *Musa *genome were between 100 kb and 500 Kb apart. The BAC end data sets for *O. sativa, A. thaliana*, maize and *M. truncatula *used for GC profiling was originally downloaded from GenBank and then the vector trimmed and cleaned sequences were downloaded from estinformatics.org [28].

### EST clustering and assembly

*Musa *EST reads was originally downloaded from GenBank and then the vector trimmed and cleaned sequences were downloaded from estinformatics.org [28] and clustered and assembled [26].

### Identification and analyses of simple sequence repeats

Perfect dinucleotide to hexanucleotide simple sequence repeats were identified using the MISA [20] Perl scripts, specifying a minimum of six dinucleotide and five tetranucleotide to hexanucleotide repeats and a maximum of 100-nucleotides interruption for compound repeats and the minimum length for mononucleotide repeats was 20 bases.

## Authors' contributions

FC conducted the bioinformatics, FC, CDT contributed to the manuscript writing, CDT managed the overall project. Both authors read and approved the final manuscript.

## References

[B1] Meinke DW, Cherry JM, Dean C, Rounsley SD, Koornneef M (1998). Arabidopsis thaliana: a model plant for genome analysis. Science.

[B2] International Rice Genome Sequencing Project (2005). The map based sequence of the rice genome. Nature.

[B3] Zhao W, Wang J, He X, Huang X, Jiao Y, Dai M, Wei S, Fu J, Chen Y, Ren X, Zhang Y, Ni P, Zhang J, Li S, Wang J, Wong GK, Zhao H, Yu J, Yang H, Wang J (2004). BGI-RIS, An integrated information resource and comparative analysis workbench for rice genomics. Nucleic Acids Res.

[B4] Lysak MA, Dolezelova M, Horry JP, Swennen R, Dolezel J (1999). Flow cytometric analysis of nuclear DNA content in Musa. Theor Appl Genet.

[B5] Kamate K, Brown S, Durand P, Bureau JM, De Nay D, Trinh TH (2001). Nuclear DNA content and base composition in 28 taxa of Musa. Genome.

[B6] Bartos J, Alkhimova O, Dolezelova M, De Langhe E, Dolezel (2005). Nuclear genome size and genomic distribution of ribosomal DNA in Musa and Ensete (Musaceae): taxonomic implications. Cytogenet Genome Res.

[B7] Arabidopsis Genome Initiative (2000). Analysis of the genome sequence of the flowering plant Arabidopsis thaliana. Nature.

[B8] Singh NK, Raghuvanshi S, Srivastava SK, Gaur A, Pal AK, Dalal V, Singh A, Ghazi IA, Bhargav A, Yadav M, Dixit A, Batra K, Gaikwad K, Sharma TR, Mohanty A, Bharti AK, Kapur A, Gupta V, Kumar D, Vij S, Vydianathan R, Khurana P, Sharma S, McCombie WR, Messing J, Wing R, Sasaki T, Khurana P, Mohapatra T, Khurana JP, Tyagi AK (2004). Sequence analysis of the long arm of rice chromosome 11 for rice-wheat synteny. Funct Integr Genomics.

[B9] Gu Y, Coleman-Derr D, Kong X, Anderson O (2004). Rapid genome evolution revealed by comparative sequence analysis of orthologous regions from four triticeae genomes. Plant Physiol.

[B10] Salse J, Piegu B, Cooke R, Delseny M (2004). New in silico insight into the synteny between rice (Oryza sativa L.) and maize (Zea mays L.) highlights reshuffling and identifies new duplications in the rice genome. Plant J.

[B11] Lai CW, Yu Q, Hou S, Skelton RL, Jones MR, Lewis KL, Murray J, Eustice M, Guan P, Agbayani R, Moore PH, Ming R, Presting GG (2006). Analysis of papaya BAC end sequences reveals first insights into the organization of a fruit tree genome. Mol Genet Genomics.

[B12] Vilarinhos AD, Piffanelli P, Lagoda P, Thibivilliers S, Sabau X, Carreel F, D'Hont A (2003). Construction and characterization of a bacterial artificial chromosome library of banana (Musa acuminata Colla). Theor Appl Genet.

[B13] SanMiguel P, Gaut BS, Tikhonov A, Nakajima Y, Bennetzen JL (1998). The paleontology of intergene retrotransposons of maize. Nat Genet.

[B14] Aert R, Sagi L, Volckaert G (2004). Gene content and density in banana (Musa acuminata) as revealed by genomic sequencing of BAC clones. Theor Appl Genet.

[B15] Yuan Q, Ouyang S, Wang A, Zhu W, Maiti R, Lin H, Hamilton J, Haas B, Sultana R, Cheung F, Wortman J, Buell CR (2005). The Institute for Genomic Research Osa1 rice genome annotation database. Plant Physiol.

[B16] The Arabidoposis Information Resource. http://www.arabidopsis.org.

[B17] Kuhl JC, Cheung F, Yuan Q, Martin W, Zewdie Y, McCallum J, Catanach A, Rutherford P, Sink KC, Jenderek M, Prince JP, Town CD, Havey MJ (2004). A unique set of 11,008 onion expressed sequence tags reveals expressed sequence and genomic differences between the monocot orders Asparagales and Poales. Plant Cell.

[B18] Wong GK, Wang J, Tao L, Tan J, Zhang J, Passey DA, Yu J (2002). Compositional gradients in Gramineae genes. Genome Res.

[B19] Santos CM, Martins NF, Horberg HM, de Almeida ER, Coelho MC, Togawa RC, da Silva FR, Caetano AR, Miller RN, Souza MT (2005). Analysis of expressed sequence tags from Musa acuminata ssp burmannicoides, var. Calcutta 4 (AA) leaves submitted to temperature stresses. Theor Appl Genet.

[B20] Thiel T, Michalek W, Varshney RK, Graner A (2003). Exploiting EST databases for the development and characterization of gene-derived SSR-markers in barley (Hordeum vulgare L.). Theor Appl Genet.

[B21] Katti MV, Ranjekar PK, Gupta VS (2001). Differential distribution of simple sequence repeats in eukaryotic genome sequences. Mol Biol Evol.

[B22] Jung S, Abbott A, Jesudurai C, Tomkins J, Main D (2005). Frequency, type, distribution and annotation of simple sequence repeats in Rosaceae ESTs. Funct Integr Genomics.

[B23] Creste S, Benatti TR, Orsi MR, Risterucci AM, Figueira A (2006). Isolation and characterization of microsatellite loci from a commercial cultivar of *Musa acuminata*. Molecular Ecology Notes.

[B24] Raboin LM, Carreel F, Noyer JL, Baurens FC, Horry JP, Bakry F, Tezenas Du Montcel H, Ganry J, Lanaud C, Lagoda PJL (2005). Diploid ancestors of triploid export banana cultivars: molecular identification of 2n restitution gamete donors and n gamete donors. Molecular Breeding.

[B25] Chou HH, Holmes MH (2001). DNA sequence quality trimming and vector removal. Bioinformatics.

[B26] Quackenbush J, Liang F, Holt I, Pertea G, Upton J (2000). The TIGR gene indices: reconstruction and representation of expressed gene sequences. Nucleic Acids Res.

[B27] Ouyang S, Buell CR (2004). The TIGR Plant Repeat Databases: a collective resource for the identification of repetitive sequences in plants. Nucleic Acids Res.

[B28] estinformatics.org. http://www.estinformatics.org.

